# Is Chronic Exposure to Raw Water a Possible Risk Factor for Amyotrophic Lateral Sclerosis? A Pilot Case-Control Study

**DOI:** 10.3390/brainsci11020193

**Published:** 2021-02-05

**Authors:** Giuseppe Stipa, Antonio Ancidoni, Monica Mazzola, Emanuela Testai, Enzo Funari, Cristina Spera, Cinzia Fanelli, Alessia Mancini, Nicola Vanacore

**Affiliations:** 1 Clinical Neurophysiology Division, Neuroscience Department, S. Maria University Hospital, 05100 Terni, Italy; c.fanelli@aospterni.it (C.F.); a.mancini@aospterni.it (A.M.); 2 National Center for Disease Prevention and Health Promotion, National Institute of Health (ISS), 34, 00162 Roma, Italy; antonio_ancidoni94@hotmail.it (A.A.); monica.mazzola@iss.it (M.M.); nicola.vanacore@iss.it (N.V.); 3 Department of Environment and Health, National Institute of Health (ISS), 299, 00161 Roma, Italy; emanuela.testai@iss.it (E.T.); enzo.funari@iss.it (E.F.); 4 Neurology Division, Neuroscience Department, S. Maria University Hospital, 05100 Terni, Italy; c.spera@aospterni.it

**Keywords:** ALS, case-control study, raw water, environmental factors

## Abstract

Background: The etiopathogenesis of amyotrophic lateral sclerosis (ALS) is still largely unknown. Methods: We performed a case-control study (33 cases and 35 controls) in Umbria, Italy. We investigated associations between common lifestyle, clinical factors, as well as environmental exposures potentially implicated with ALS onset. Face-to-face interviews were carried out. All cases were recruited and diagnosed according to El Escorial criteria. Case-control comparisons were made for educational and residential status, occupational exposures, and clinical and lifestyle factors prior to cases’ dates of diagnosis. Results: Our results showed an increased risk of ALS for subjects chronically exposed to raw water use (odds ratio (OR) = 6.55, 95% confidence interval (CI): 2.24–19.12). Garden activities showed a tight association with ALS as well, very likely as a consequence of chronic raw water exposure. Indeed, we could exclude an impact for pesticides, as no significant differences were observed in pesticide exposure in the two groups interviewed. However, cases were more often exposed to fertilizers. After adjustment for age, sex, and heavy physical activities, exposure to raw water was still associated with increased ALS risk (OR = 4.74, 95% CI: 1.33–16.85). Discussion: These findings suggest an association between ALS and exposure to raw water, which should be further investigated for the presence of chemicals interfering with nervous system functionality.

## 1. Introduction

Amyotrophic lateral sclerosis (ALS) is a neurodegenerative disease (ND), and the most common motor neuron disease [1]. It is fatal and characterized by highly progressive neurodegeneration, affecting both superior and inferior motor neurons in several regions of the spinal cord and brain stem. In 90–95% of the cases, ALS is sporadic, in only 5–10% is it hereditary, known as familial ALS (FALS) [2,3]. The median age at onset is 58–63 years for sporadic ALS (SALS), and around 47–52 years in inherited cases [4].

At the onset, muscle fatigue, fasciculations, and muscle cramps are considered just some of the major symptoms of motor neuron impairment; however, swallowing, tongue dysfunction, and increased salivation are also known symptoms of a motor neuron impairment in the brain stem and consequently in the central nervous system (CNS). Nutritional status and respiratory failure are associated with disease severity and death usually occurs within 3–4 years.

Recent population-based studies reported an ALS prevalence between 4.1 and 8.4 per 100,000 population, with male sex considered a risk factor and the male-to-female risk ratio between 1 and 2 [5,6], with few studies reporting a risk ratio even 3-fold higher in men [7]. Some population-based studies reported an ALS incidence in Europe at 2.16 per 100,000, while this data is not yet known worldwide [4]. Several studies reported that physical exercise, connected with head injuries, especially in professional contact sports, is a risk factor for ALS and more generally for NDs (e.g., Alzheimer’s and Parkinson’s diseases) [8,9,10].

Furthermore, some epidemiologic studies focused on the role of environmental factors suspected to be linked to ALS, including solvents, heavy metals, and pesticides. A recent systematic review [11] showed that lead exposure increased the risk of ALS and Parkinson’s disease. Furthermore, a case-control study conducted in New England between 1993 and 1996 suggested that lead exposure could play a crucial role in ALS etiology [12]. Selenium and arsenic, possibly present in groundwater and well water as contaminants, have also been considered as risk factors for SALS [13,14].

Demographic and disease-related data have been used to examine the relationship between a rural environment and ALS risk: several studies suggested that ALS is more frequently diagnosed in inhabitants living in rural areas [15]. Although a rural residence itself does not give rise to a statistically significant risk of ALS, subjects chronically exposed to a high concentration of pesticides in agricultural settings show a higher risk for ALS than the general population [16,17,18]. Recently, in a rural context, exposure to grass and well water for private use has been considered as a key factor in ALS onset, suggesting a high concentration of agricultural chemicals, neurotoxic factors, or mycotoxins as potential etiology of sporadic ALS [19,20,21]. Finally, in a case-control study in New England, with 295 patients and 225 controls, patients reported having lived full-time within two miles of a water body more frequently than did controls (OR 1.59) [22].

At present, none of the environmental factors has been conclusively and causally associated with ALS. The aim of this pilot case-control study was to investigate possible associations between common lifestyle, clinical factors, and environmental exposures potentially implicated with ALS onset in a province of a Central Italian region.

## 2. Methods

### 2.1. Study Design, Patients Characteristics, and Residential Exposure

This study was performed in Umbria, a region in central Italy in the province of Terni. Sixty-eight participants (33 cases and 35 controls) were enrolled between March 2016 and February 2020 at the Clinical Neurophysiology division in the S. Maria Hospital in Terni. All controls (age ≥ 18) were subjects without a known history of neuromuscular and neurodegenerative pathologies recruited at the same Neurological Centre of Terni hospital for electroencephalogram (EEG) examination, mainly for suspected convulsion condition. All 33 patients were selected and categorized as definite, probable, possible, and suspected ALS diagnosis according to El Escorial criteria [23]. They were invited to answer a standardized questionnaire, in which subjects were asked for their date, birthplace, and place of residence. All questionnaires were gathered by qualified members of the hospital clinical staff. According to demographic data from the Italian Institute of Statistics (ISTAT), we identified three levels of urbanization in our study: <20,000 inhabitants, between 20,000 and 50,000, and >50,000. We stratified cases and controls for the municipality of residence: we aimed to control the living environment as a possible confounding factor to avoid selection bias. For all cases and controls, residential exposure was evaluated: Terni province is known to be a highly productive site with several industries (e.g., steel mill, textile and chemical industries, and farms). Subjects that answered to live or have lived close to any of the aforementioned production sites in the past were considered potentially exposed to polluted air and water.

Written informed consent was gathered from all participants. For patients and controls who were not able to give their informed consent due to mental or physical disability, this was provided by a family member or legal representative of the participant. We would specify that in this study we acquired more detailed information on the history of cases and controls related to their residence and lifestyle habits. Protection of personal data from all participants was ensured in accordance with Regulation (EU) 2016/679 of the European Parliament and of the Council of 27 April 2016 [24].

### 2.2. Lifestyle Factors, Occupational Exposures and Medical Histories

Information was taken on smoking and dietary habits, alcohol, and other daily habits during their lifetime as well as their knowledge about possible exposure to any chemicals, waste, or toxic agents. Regarding information on the occupational activities, we defined the longest during the subject’s lifetime as the main one, but all different occupations were considered, to also identify temporary exposures potentially relevant as ALS risk factors. We stratified the occupational exposure as “not exposed”, “possibly exposed”, and “probably exposed”: subjects employed in administrative jobs where a heavy physical activity was not required or where no exposure to chemicals related to ALS (solvents, pesticides, or metals) were considered as not exposed to ALS risk; subjects whose jobs required heavy physical activity or exposure to pesticides, solvents, and metals, known to be neurotoxic, were considered possibly or probably exposed to ALS risk. Information was taken about clinical histories, drug consumption, and physical traumas (especially head/neck injuries). Past medical history and possible NDs in family history for each case were also assessed to investigate possible genetic influences.

### 2.3. Exposure to Raw Water and Hobby Activities

Raw water is water found in the environment that has not been treated to remove any chemicals, toxins, bacteria, or spores. Raw water includes rainwater, groundwater, well water, and water bodies (e.g., lakes and rivers). Treated water means all treatments to make the water suitable for human use, regardless of the source, such as filtration and use of chemicals (e.g., chlorine) and physical (e.g., UV radiations) disinfectants, according to Ministerial Order 7 February 2012, n. 25 [25]. Detailed information about untreated water or well water use was collected: enrolled subjects were asked to specify the reason why untreated water was used. Finally, hobby activities were evaluated in this study: all participants were asked which activities were practiced the most with the aim to identify ones related to untreated water uses (e.g., gardening or family vegetable production with consumption of self-produced food). Pesticides and fertilizers utilization was also investigated since other studies have hypothesized the possible neurotoxicity of these products.

### 2.4. Statistical Analysis

Statistical analysis was used to calculate the odds ratio (OR) and associated 95% confidence interval (CI) were estimated to estimate ALS risk factors. The T-student test was used for continuous variables and the Chi-square test for categorical variables. The Shapiro–Wilk test for normality was used. Considering the explorative role of this study, we did not correct for multiple comparisons. A logistic regression model was performed including as independent variable those statistically significant at univariate analysis and as dependent variable the condition of case or control. We included sex, age, and educational level as confounding factors in the multivariate analysis. Considering the exploratory aim of our study the Bonferroni method was not used. The software used for data analysis was SPSS 26.0. In our study, results were considered statistically significant for *p* values < 0.05.

## 3. Results

### 3.1. Study Design, Patients Characteristics, and Residential Exposure

In total, 33 cases (M/F: 24/9) and 35 controls (M/F: 23/12) were enrolled and characterized on the basis of information collected by means of the questionnaire administered as a face-to-face interview (Table 1). In detail, 59 subjects (86.8%) were able to perform a personal interview. For nine participants who could not support verbal communication, the interview was carried out with the support of their family members. The mean age in cases and controls was 67.58 ± 10.77 and 66.63 ± 11.42, respectively. According to the El Escorial criteria [23], 21 cases were diagnosed as definite ALS, 2 cases as probable ALS, 8 as possible ALS, and 2 cases as suspected ALS. ALS median age-onset was 60.75 (±11.54). Among 33 cases, 3 (9.1%) declared having at least one relative affected by ALS, another 8 subjects reported having a relative affected by other NDs, whereas 20 patients declared to have never had a relative affected by any ND. Twenty patients (60.6%) were being treated with Riluzole, a drug for ALS treatment, at the time of the interview. Regarding educational level, only three controls were graduated, while homogeneous distribution between cases and controls was observed among subjects with a high school diploma or lower as shown in Table 1 (*p* = 0.41).

Cases and controls were matched by choosing from the three categories of municipality residence: among controls, 13 (37.1%) were residents in municipalities of <20,000 inhabitants, 18 (51.4%) subjects lived in municipalities with >50,000 inhabitants, and the remaining 4 (11.4%) lived in municipalities with intermediate population density. Regarding cases, 11 (33.3%) used to live in municipalities with >50,000 inhabitants, 15 (45.5%) lived in municipalities with <20,000 inhabitants, and again only 4 (12.1%) lived in municipalities with an intermediate number of inhabitants. No statistical significance regarding residence was found (Table 1). Furthermore, we did not observe differences between cases and controls regarding the proximity of residences to industrial sites (data not shown).

### 3.2. Lifestyle Factors, Occupational Exposure, and Medical Histories

As shown in Table 2, smoking as a risk factor for ALS was evaluated: considering current smokers and former smokers as a single variable, we estimated an OR = 1.07 (95% CI: 0.37–3.09), not statistically significant. Alcohol consumption was not associated with a higher risk of ALS onset as well. Regarding dietary habits, the majority of participants followed an omnivorous diet: all subjects were asked consumption frequency of several food groups. However, from the statistical analysis we could not find any significant association between any food group consumption and ALS risk (data not shown). Carrying out heavy physical activities related to the occupational setting was evaluated: we observed an increased risk for ALS patients (OR = 2.77, 95% CI: 1.00–7.63), but sports activities were not associated with any increased ALS risk. Our data regarding the occurrence of a physical trauma indicate a potential for a higher ALS risk but without any statistical significance (OR = 1.49, 95% CI: 0.55–4.03, *p* = 0.45). This could be due to the very similar number of head injuries reported by cases and controls (eight and seven respectively). It is worthy of note that two ALS patients reported they have suffered more than one head injury throughout their lives.

### 3.3. Exposure to Raw Water and Pesticides

The most robust association was found between raw water utilization and an increased risk of ALS (OR = 6.55, 95% CI: 2.24–19.12). Among the 33 patients, 25 made use of untreated water during their lifetime for several years: average time observed for raw water utilization was 28.1 ± 13.1 years (range: 10–50), most commonly for plant irrigation and private drinking water supply. In 22 of the cases that used raw water during their life, they reported having never had relatives with ALS in their family history. Among three familial ALS cases, two made use of raw water in their life mainly for gardening and watering vegetables, while one declared to have never used raw water. In controls, only 11 subjects made use of raw water during their lifetime. Information on average time spent using raw water was available only for eight subjects (26.1 ± 17.2 years (range: 10–60)). In the present study, we also observed an association, although not statistically significant, between well water consumption and ALS risk. Unfortunately, raw water exposure could not be quantified (or even estimated), due to heterogeneity of the reported activities, some dated to many years before the interview.

As lifestyle factors included in this study, cultural interests, hobbies, and social activities were also evaluated: in our study we observed that 30/33 patients currently have a hobby or had one in the past, while only 17/35 control subjects claimed to have a hobby (OR = 6.82, 95% CI: 2.17–25.94). But the most relevant information was the evaluation of which kind of hobbies they have practiced: watering plants, harvesting, consuming home-grown vegetables, and picking fruits or vegetables were the most frequent activities. Indeed, a high number of subjects exposed to untreated water (e.g., well water and underground water tanks) made use of it mainly for gardening and home farming, activities showing a significant association with ALS risk (OR = 6.22, 95% CI: 1.45–26.64). It is noteworthy that subjects exposed to raw water had spent on average 10 h a week for 31.3 ± 14.1 years (range: 10–50) mostly during the dry season when rains are less frequent, and irrigation is necessary. After adjustment for age, sex, heavy physical activities, and hobbies, exposure to raw water was still associated with increased ALS risk (OR = 4.74, 95% CI: 1.33–16.85). We did not observe any correlation between ALS age-onset and years of exposure to raw water (*p* = 0.77), however, this information is based only on ten subjects for which we had this information. We evaluated whether there was any association between ALS definite (n = 20) diagnosis and other ALS diagnosis (n = 13) and years of exposure to raw water, however, the difference was not statistically different (*p* = 0.58). Furthermore, we did not observe differences in pesticide utilization among cases and controls while 24.2% of the cases vs. 0% of the controls were exposed to fertilizers (*p* = 0.02) (Table 2).

## 4. Discussion

We performed a pilot case-control study by recruiting subjects in the province of Terni (Umbria) in Central Italy. After stratification for the number of inhabitants for the municipality of residence and after evaluating possible residential exposure to any industrial sites, we did not observe any association with living setting and ALS risk. However, some epidemiologic studies have reported a higher ALS risk for people living in rural areas than in urbanized districts [16].

No differences were found in our study in regard to educational attainment: however, in some studies, a high level of education is both negatively [17,26] and positively associated with ALS risk [27]. The negative association can be explained since white-collar workers, often associated with a high level of education, perform their work sitting at a desk, this being negatively correlated with physical traumas and head injuries, which are considered risk factors for ALS, when compared to blue-collar workers. In fact, heavy physical activities associated with work showed a positive and significant association with ALS in our study. This result is in line with data from a recent meta-analysis showing a relative ALS risk of approximately 30% in subjects that, during their lifetime, chronically experienced heavy physical work [11]. A positive, but not statistically significant association (*p* = 0.45) was found between physical traumas and ALS risk in our study and despite some controversy in recent years, the literature seems to support this association, mostly related to professional contact sports [8,9,10,28].

In line with several other studies [17,29,30], where relative risks or odds ratios observed were approximately 1, no association between smoking habits and ALS risk was found, considering “current smokers” and “former smokers” as a unique group. The issue is very controversial, since other reports [31] showed an association between smoking cigarettes and ALS risk, higher in former smokers, although other authors reported a stronger association in current smokers [32]. The reason for these discrepancies in literature is yet to be understood.

This study has not found any differences with dietary habits among patients and controls: all participants reported that they have generally followed a regular Mediterranean diet, which was indicated in a recent literature review as an important defense factor due to its antioxidant power, improving the resilience to pathogenic mechanisms related to diseases [33].

The most robust association evidenced in our study is the one between ALS and raw water use, which is expected to be higher in rural areas and small municipalities (Table 3). Recently, Filippini et al. [19] in a study conducted in Italy on 95 cases and 135 controls, suggest an association, although not significant, between well water consumption for drinking water and ALS risk in the Northern Italian population (OR = 3.33, 95% CI: 0.31–35.69) but not in Southern Italy, where a stronger association with ALS risk was found with current well water consumption for irrigation (OR = 1.74, 95% CI: 0.18–17.21). We observed a not statistically significant association in this study as well, between well water consumption and ALS. Geographical localization of ALS clusters has been previously associated with lake water quality [13,14] and, according to a study in the USA [20], well water used for drinking might be associated with sporadic ALS. In literature, it is widely reported that there is a statistically significant association between ALS onset and well water consumption or water uses from rivers, lakes, or streams in several ecological studies [20,22,34]. Moreover, other authors speculated on the association between ALS and activities related to raw water use (e.g., gathering, fishing, use of well water) in very different living environments from our study: in a survey in Papua, Indonesia, between 2001 and 2012, Okumiya et al. [35] highlighted that neurodegenerative diseases such as ALS and parkinsonism were detected mostly in villages nearby three rivers: thus they hypothesized that those diseases may be somehow associated with the use of shallow wells. In all cases, the exposure to raw water was not quantified, as well, the potential agent and the pathogenetic mechanisms were not identified to confirm the biological plausibility and the causality of these associations. However, pathogenetic mechanisms to confirm these associations are yet to be understood. To our knowledge, this is the first population-based case-control study to report an association between raw water utilization and ALS risk. Our data support the hypothesis of raw water use as a potential risk factor for ALS. In fact, although we are aware of the limited number of enrolled patients, this study shows that nearly 73% of ALS patients declared to have used raw water for a long time during their lifetime: watering for farming and gardening were the main activities carried out. It is noteworthy that analyzing patient’s family histories; we identified three patients (9.1%) with an ALS-affected relative, suggesting for them a genetic influence with a percentage similar to what is described in the literature for hereditary ALS [3,4]. Two out of three patients affected by hereditary ALS declared to have continuously used untreated water leading us to hypothesize that raw water might have increased the risk of developing ALS [36,37]. It is well known that untreated water could be a source for several potential neurotoxic factors: mycotoxins, cyanobacteria, fertilizers or pesticides, and other substances (e.g., metals or solvents) for which many epidemiological studies have highlighted associations with neurotoxic effects and consequent higher incidence of ALS [13,14,20,38,39,40]. Filippini et al. [19] observed an odds ratio of 1.95 (95% CI: 0.88–4.36) for use of herbicides in gardening associated with ALS risk while results on pesticide utilization offered little support of increased ALS risk (OR = 1.15, 95% CI: 0.56–2.39). Furthermore, a meta-analysis [41] highlighted a significant association between pesticide exposure and risk of ALS (OR = 1.88, 95% CI: 1.36–2.61). However, our results on a small group of patients did not confirm this association regarding pesticide utilization, but we observed an increased exposure to fertilizers in cases. This leads us to hypothesize that in our study the association with ALS onset is closely related to raw water use.

This study, however, has some limitations. Firstly, cases and controls were identified in a restricted geographical area, thus avoiding differences between clinic and hospital-based studies, as well as environmental factors that might be region and territory-specific. The enrollment of ALS-affected patients with different clinical stages, possibly reflecting the clinical issues of the disease diagnosis, although taken as an apparent limitation by some authors, was considered as an expression of current clinical practice. As a limitation, the number of subjects included in this study is low. The reason was due to the rare frequency of the disease in the relatively small population of the province of Terni (about 225,000 inhabitants). Unfortunately, the sample size was too small to carry out a subtype analysis. Phenotypic subtypes of ALS were not investigated in this study, only environmental factors related to ALS onset. Unfortunately, as a limitation of this study, we did not collect data regarding family history in controls. Although we aimed to include subjects able to answer to a questionnaire through a personal interview, we could not exclude patients whose questionnaires were filled in by relatives; the reason was to avoid the reduction of our population and weakening of our results.

In addition, raw water exposure was not quantified, as well as the quality of raw water, in terms of potential toxic agent(s) content. We considered, however, that the present status of water cannot be taken as representative of the exposure experienced by the enrolled individuals over time (sometimes starting many years before this study was conducted) and therefore cannot be used as a robust “proxy” of the actual exposure. Given the characteristics of our pilot study in a small group of cases, we felt it was unnecessary to carry out further statistical analyzes by diagnostic level and clinical ALS subtypes as we would have increased the probability of making a false positive error. For these methodological and statistical reasons, potential sub-phenotypes or dynamics in the clinical course were not considered in our study. The identification of the potential toxic agent(s) [42,43,44,45] in terms of plausibility and mechanism of action will be the object of future studies.

## 5. Conclusions

A multicentric study would be useful to enroll a higher number of subjects to further investigate the role of risk factors related to the use of raw water as well as possible region-specific or environment-specific factors. So far we have not analyzed any chemicals (single or as a mixture), toxins, or bacteria associated with raw water in the setting where the study was carried out. Once the association between raw water and the onset of ALS is strengthened by a larger study, it would be necessary to investigate not only water quality by looking at some selected contaminants and trying to make estimations over time, but also possible genetic factors that could synergize with environmental ones giving rise to gen-environment interactions.

## Figures and Tables

**Table 1:** Demographical characteristics, occupational and residential exposure of study population.

Characteristics	Cases *n* = 33 (%)	Controls *n* = 35 (%)	Total *n* = 68 (%)	*p*
**Sex**				0.53 **
**Male**	24 (72.7)	23 (65.7)	47 (69.1)	
**Female**	9 (27.3)	12 (34.3)	21 (30.9)	
**Age**				0.93 *
**Mean (SD) years**	67.58 (10.77)	66.63 (11.42)	67.07 (10.99)	
**Municipality ^†^**				0.48 **
**>50,000**	11 (33.3)	18 (51.4)	29 (42.6)	
**50,000–20,000**	4 (12.1)	4 (11.4)	7 (10.3)	
**<20,000**	15 (45.5)	13 (37.1)	29 (42.6)	
**Education level**				0.41 **
**Primary school**	7 (21.2)	7 (20)	14 (20.9)	
**Middle school**	12 (36.4)	12 (34.3)	24 (35.8)	
**High school**	13 (39.4)	13 (37.1)	26 (38.8)	
**College or higher**	0 (0.0)	3 (8.6)	3 (4.5)	
**Residential exposure ^§^**				0.34 **
**Not exposed**	7 (21.2)	11 (31.4)	18 (26.5)	
**Exposed**	26 (78.8)	24 (68.6)	50 (73.5)	
**Occupational exposure** **^ǂ^**				0.41 **
**Not exposed**	11 (33.3)	16 (45.7)	27 (39.7)	
**Possibly exposed**	13 (39.4)	9 (25.7)	21 (30.9)	
**Probably exposed**	7 (21.2)	9 (25.7)	16 (23.5)	

^†^ Data reported referring to numbers of inhabitants per municipality. Municipalities were divided into three categories: less than 20,000, between 20,000 and 50,000, more than 50,000 inhabitants. ^§^ Results have been categorized as “not exposed” and “exposed” on the basis of the proximity of the place of residence to industrial and production sites and possible exposure to air and water pollutants. ^ǂ^ Subjects employed in administrative jobs or for which heavy physical activities were not required or not exposed to chemicals have been considered as “not exposed” to ALS risk factors. Subjects whose jobs required heavy physical activity or exposure to chemicals were considered “possibly exposed” or “probably exposed” to ALS risk factors. * T-student test for continuous variables: Age (t = −0.35; df 66). ** Chi-square for categorical variables: Sex (χ = 0.39; df 1); Municipality (χ = 1.46; df 2); Educational level (χ = 2.87; df 3); Residential exposure (χ = 0.91; df 1); Occupational exposure (χ = 1.77; df 2).

**Table 2:** Odds ratio (OR) with 95% confidence interval (95% CI) of ALS risk associated with lifestyle factors.

Factor	Cases *n* = 33 (%)	Controls *n* = 35 (%)	OR	95% CI	*p*
**Smoking**			1.07	0.37–3.09	0.91
**Yes**	24 (72.7)	23 (65.7)			
**No**	9 (27.3)	10 (28.6)			
**Alcohol**			1.61	0.35–7.36	0.54
**Yes**	29 (87.9)	30 (85.7)			
**No**	3 (9.1)	5 (14.3)			
**Heavy physical activities**			2.77	1.00–7.63	0.05
**Yes**	19 (57.6)	12 (34.3)			
**No**	12 (36.4)	21 (60)			
**Physical traumas**			1.49	0.55–4.03	0.45
**Yes**	19 (57.6)	17 (48.6)			
**No**	12 (36.4)	16 (45.7)			
**Sport**			1.59	0.61–4.17	0.34
**Yes**	17 (51.5)	14 (40)			
**No**	16 (48.5)	21 (60)			
**Hobby**			6.82	1.96–23.78	0.001
**Yes**	29 (88.2)	17 (48.6)			
**No**	4 (11.8)	16 (45.7)			
**Use of pesticides**			0.63	0.14–3.30	0.23
**Yes**	4 (12.1)	3 (8.6)			
**No**	29 (87.9)	32 (91.4)			
**Use of fertilizers**			-	-	0.02
**Yes**	8 (24.2)	0 (0.0)			
**No**	25 (75.8)	35 (100)			
**Use of raw water**			6.55	2.24–19.12	<0.001
**Yes**	24 (72.3)	11(31.4)			
**No**	8 (24.2)	24 (68.6)			
**Use of any private well**			2.64	0.59–11.83	0.20
**Yes**	19 (57.6)	6 (17.1)			
**No**	6 (18.2)	5 (14.3)			

There are missing data for some of the variables, percentages do not correspond to the total. For some questions, not all participants answered adequately.

**Table 3:** Multivariate regression analysis of amyotrophic lateral sclerosis (ALS) risk factors.

Variable	B	S. E.	Wald	OR	95%CI	*p*
**Sex**	0.36	0.68	0.28	1.44	0.38–5.44	0.59
**Age**	0.002	0.03	0.003	1.00	0.95–1.06	0.96
**Heavy physical activities**	1.47	0.68	4.75	4.35	1.16–16.35	0.03
**Hobby**	2.04	0.79	6.70	7.71	1.64–36.16	0.01
**Raw water**	1.56	0.65	5.78	4.74	1.33–16.85	0.02
